# High *zT* and Its Origin in Sb‐doped GeTe Single Crystals

**DOI:** 10.1002/advs.202002494

**Published:** 2020-11-06

**Authors:** Ranganayakulu K. Vankayala, Tian‐Wey Lan, Prakash Parajuli, Fengjiao Liu, Rahul Rao, Shih Hsun Yu, Tsu‐Lien Hung, Chih‐Hao Lee, Shin‐ichiro Yano, Cheng‐Rong Hsing, Duc‐Long Nguyen, Cheng‐Lung Chen, Sriparna Bhattacharya, Kuei‐Hsien Chen, Min‐Nan Ou, Oliver Rancu, Apparao M. Rao, Yang‐Yuan Chen

**Affiliations:** ^1^ Institute of Physics Academia Sinica Taipei 11529 Taiwan, ROC; ^2^ Dept. of Engineering and System Science National Tsing Hua University Hsinchu 30013 Taiwan, ROC; ^3^ Taiwan International Graduate Program Taipei 115 Taiwan, ROC; ^4^ Clemson Nanomaterials Institute Department of Physics and Astronomy Clemson University Clemson SC 29634 USA; ^5^ Air Force Research Laboratory WPAFB Dayton OH 45433 USA; ^6^ National Synchrotron Radiation Research Center Hsinchu 30077 Taiwan, ROC; ^7^ Institute of Atomic and Molecular Sciences Academia Sinica Taipei 10617 Taiwan, ROC

**Keywords:** energy generation, four‐phonon decay, GeTe, phonon dispersion, thermoelectric materials

## Abstract

A record high *zT* of 2.2 at 740 K is reported in Ge_0.92_Sb_0.08_Te single crystals, with an optimal hole carrier concentration ≈4 × 10^20^ cm^−3^ that simultaneously maximizes the power factor (*PF*) ≈56 µW cm^−1 ^K^−2^ and minimizes the thermal conductivity ≈1.9 Wm^−1^ K^−1^. In addition to the presence of herringbone domains and stacking faults, the Ge_0.92_Sb_0.08_Te exhibits significant modification to phonon dispersion with an extra phonon excitation around ≈5–6 meV at *Γ* point of the Brillouin zone as confirmed through inelastic neutron scattering (INS) measurements. Density functional theory (DFT) confirmed this phonon excitation, and predicted another higher energy phonon excitation ≈12–13 meV at *W* point. These phonon excitations collectively increase the number of phonon decay channels leading to softening of phonon frequencies such that a three‐phonon process is dominant in Ge_0.92_Sb_0.08_Te, in contrast to a dominant four‐phonon process in pristine GeTe, highlighting the importance of phonon engineering approaches to improving thermoelectric (*TE*) performance.

## Graphical abstract

The origin of high *zT* ≈ 2.2 at 740 K of Ge_0.92_Sb_0.08_Te is uncovered via DFT and inelastic neutron scattering measurements that reveal the presence of a new phonon band at a transfer energy *E* ≈5–6 meV, highlighting the importance of phonon engineering approaches to improving thermoelectricperformance.

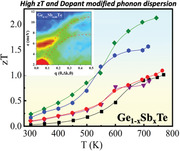

## 1 Introduction

Bulk GeTe, a IV–VI chalcogenide, exhibits a wide range of intriguing fundamental properties of technological importance that include: i) phase‐change properties for optical data storage applications as in (GeTe)*_m_*(Sb_2_Te_3_)*_n_*,^[^ ^1^, ^2^ ^]^ ii) ferroelectricity in bulk and nanoscale crystals,^[^ ^3^ ^]^ iii) Rashba spin splitting for spintronics applications,^[^ ^4^ ^]^ and iv) a renewed interest in thermoelectric (TE) conversion of “waste heat” to electricity.^[^ ^5^, ^6^, ^7^ ^]^ With respect to iv), the implementation of TE energy conversion devices on a commercial scale has been challenging due to the strong coupling between TE material properties.^[^ ^8^ ^]^ The coupled Seebeck coefficient *S*, electrical conductivity *σ* and thermal conductivity *κ* give rise to a TE conversion efficiency denoted by a dimensionless figure‐of‐merit, or$zT=\frac{S^{2}\sigma T}{\kappa }.$ Here *κ* (= *κ*_*e*_ + *κ*_*lat*_) is the sum of the electronic (*κ*_*e*_) and lattice (*κ*_*lat*_) thermal conductivities. Several strategies to enhance *zT* include i) enhancing the TE power factor *PF* ( = *S* ^2^/*ρ*) via energy filtering of charge carriers,^[^ ^9^, ^10^ ^]^ or introduction of resonance levels in the valence or conduction band to improve *S*,^[^ ^11^ ^]^ and/or reduce *ρ* (= 1/*σ*) through band‐structure engineering^[^ ^12^, ^13^ ^]^ and modulation doping,^[^ ^14^ ^]^ and ii) reducing *κ*_*lat*_ through phonon engineering. This can be achieved by the incorporation of point defects,^[^ ^15^ ^]^ stacking faults,^[^ ^16^ ^]^ dislocations,^[^ ^17^, ^18^ ^]^ vacancies,^[^ ^19^ ^]^ nanostructuring,^[^ ^20^, ^21^ ^]^ nanocomposites,^[^ ^22^ ^]^ secondary phase precipitates,^[^ ^23^ ^]^ and phase separation techniques,^[^ ^24^ ^]^ all resulting in enhanced phonon scattering. Low intrinsic *κ*_*lat*_ is also attributed to phonon anharmonicity in single‐crystalline SnSe, and is responsible for its promising TE properties.^[^ ^25^, ^26^ ^]^

GeTe belongs to the same family of chalcogenides such as PbTe and SnSe, which has been of interest since 1960s with a recent report of *zT* ≈ 1.85 at 725 K with Sb‐doping in Ge_1−_ *_x_*Sb*_x_*Te crystalline ingots.^[^ ^27^ ^]^ Pristine GeTe exhibits a high hole carrier density ≈ 10^21^ cm^−3^ due to its intrinsic Ge vacancies, consistent with its low *S* ≈ 30 µV K^−1^, high *σ* ≈ 8500 S cm^−1^ and a high *κ* ≈ 8 W m^−1^ K^−1^ comprising of a large *κ*_*e*_ contribution. When doped with Sb, GeTe simultaneously exhibits: i) a suppressed high *p*‐type carrier concentration that enhances *S*, and ii) an enhanced point defect phonon scattering to reduce *κ*_*L*_, leading to a *zT* ≈ 1.8 at 725 K in Ge_1−_ *_x_*Sb*_x_*Te (*x* = 0.10) and Ge_1−_ *_x_*_−_ *_y_*Bi*_x_*Sb*_y_*Te (*x* = 0.05, *y* = 0.10) crystalline ingots and composites, respectively.^[^ ^27^, ^28^ ^]^ Recently, we also reported a high *zT* ≈ 1.9 in Bi‐doped GeTe single crystals, which was brought by lowering the thermal conductivity.^[^ ^29^ ^]^ In addition to single crystals, studies have reported *zT* values ranging from 2.2–2.4 polycrystalline GeTe doped with Bi, In, Zn, Pb, and Sb.^[^ ^30^, ^31^, ^32^ ^]^

While high *zT* values have been reported in both single‐ and poly‐crystalline GeTe, its microscopic origin has not been explored systematically. By engineering defects into crystalline materials through controlled doping, *κ*_*lat*_ can be lowered through increased point scattering and anharmonicity‐driven Umklapp scattering.^[^ ^33^ ^]^ Here, in order to probe dopant‐influenced phonon scattering and its effect on lowering *κ*_*lat*_, we conducted systematic transport, inelastic neutron scattering (INS), and spectroscopic studies on high quality single‐crystalline GeTe doped with varying amounts of Sb. Our single‐crystal Ge_1‐x_Sb_x_Te samples (henceforth referred as GST) exhibited the highest *zT* (≈2.2 at 740 K for Ge_0.92_Sb_0.08_Te) reported to date in GST single crystals. In addition to a high *PF*, whose origins have been discussed previously,^[^ ^27^ ^]^ our GST samples exhibited record low thermal conductivities (*κ*_*lat*_ ≈ 0.46 Wm^−1^ K^−1^ at 740 K). Notably, the single‐crystalline nature of our GST allowed us to unravel the fundamental processes that underpin thermal conductivity, which cannot be elicited from polycrystalline GST. The INS measurements on GST crystals revealed a Sb‐induced modified phonon dispersion with an extra phonon excitation at a transfer energy *E* ≈ 5–6 meV near the *Γ* point. Density functional theory (DFT) confirmed this phonon excitation, and predicted another higher energy phonon excitation ≈12–13 meV at the *W* point which was not verified by INS. The Sb‐dopant added extra states in the phonon density of states, increasing the phonon scattering rates and lowering the thermal conductivity in GST, which was confirmed by modeling both our measured thermal conductivity data and the temperature‐dependent polarized Raman spectra.^[^ ^34^ ^]^ The decrease in *κ*_*lat*_ was found to be predominantly caused by a three‐phonon decay process in GST unlike the four‐phonon decay process that is dominant in pristine GeTe.

## 2 Results and Discussion

### 2.1 Crystal Structure and Phase Purity

**Figure 1:**
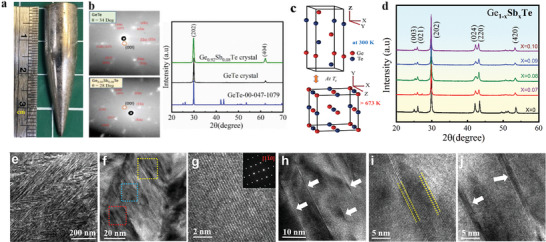
Characterization of Sb‐doped GeTe single crystals (GST). a) Image of an as‐prepared GeTe single crystal. b) Laue diffraction and X‐ray diffraction (XRD) patterns of pristine GeTe and Ge_0.92_Sb_0.08_Te crystals with suggested plane indices in red. The red circle near the center of the diffraction pattern represents the *<00l>* planes. The tilt‐angles of *c*‐axes to crystal growth direction are identified as ≈34° and ≈28° for the pristine GeTe and Ge_0.92_Sb_0.08_Te crystals, respectively. c) Schematic of the crystal structures at room temperature and above the phase transition temperature. d) Room temperature powder XRD patterns of Ge_1‐_ *_x_*Sb*_x_*Te (*x* = 0, 0.07, 0.08, 0.09, 0.10). Electron microscope images of Ge_0.92_Sb_0.08_Te: e) low magnification TEM image; f) HRTEM image of domain structures; g) HRTEM image in a defect free region with corresponding SAED pattern shown in the inset; h) Arrows highlight the nanoscale planar defect layers in the yellow box shown in (f); i) Vacancy layer from the blue boxed region in (f); j) Stacking faults images from the red boxed region in (f).

**Figure** **1**a shows an optical image of single crystalline GeTe (≈20 g) grown by the Bridgman method. The Laue diffraction patterns in Figure 1b show the diffraction peaks from various planes in single crystals of GeTe and Ge_0.92_Sb_0.08_Te. The *c*‐axis of the crystal is tilted by 34° and 28° for GeTe and GST with respect to the growth direction, respectively (Figure S1, Supporting Information). In contrast to sharp diffraction spots exhibited by GeTe, a slight astigmatism of the diffraction spots of Ge_0.92_Sb_0.08_Te indicates the presence of short‐range ordering defects in the GST crystal created by Sb dopants. Nevertheless, the sharp diffraction peaks in Figure 1b indicate the high quality of the single crystals without any impurities. The corresponding crystal structures of GeTe at room temperature and the high temperature phases are shown schematically in Figure 1c. Bulk GeTe undergoes a second‐order phase transition from a ferroelectric rhombohedral *α*‐GeTe (space group *R*3*m*) to a high temperature cubic paraelectric *β*‐GeTe (space group $Fm\bar{3}m$) at a critical temperature, *T*_c_ ≈ 650 ± 100 K. While the *β*‐GeTe phase exhibits the lattice parameters *a* = 6.020 Å, *α*‐GeTe exhibits the lattice parameters *a* = *b* = 4.1719 Å and *c* = 10.710 Å arising from a distorted NaCl type structure with an angular distortion ≈1.65° in the unit cell along the [111] direction.^[^ ^35^, ^36^ ^]^ The room temperature powder x‐ray diffraction (XRD) patterns of GST (*x* = 0, 0.07, 0.08, 0.09, 0.10) crystals are shown in Figure 1d, and all the observed diffraction peaks can be indexed to the rhombohedral structure (space group, *R3m*) with no detectable impurity or foreign phases. The doublet present between the angles 2*θ* ≈ 41°–45° in pristine GeTe corresponds to the (024) and (220) diffraction peaks of the rhombohedral phase, and tends to merge into a single peak with increasing Sb (0%–10%) doping indicating that the Sb dopant drives the shift towards to the cubic phase ($Fm\bar{3}m$). Overall, the shift of the (202) peak (Figure 1b) towards lower angles (2*θ* ≈ 29.88°) with increasing Sb content could be attributed to the relative size differences between the Ge and Sb atoms. A linear increase in the lattice constants *a* and *b* is observed with increasing Sb dopant concentration in GST (Figure S2, Supporting Information); this is in contrast to the behavior of the *c*‐axis lattice constant, which decreases linearly indicating homogeneous solid solubility of Sb doping in GeTe. In addition, Sb‐doping in GeTe also tends to shift the *T_c_* to lower temperatures as observed from the shift in the characteristic heat capacity *C*_p_ peak towards lower temperatures compared to that of the pristine GeTe. (Figure S3, Supporting Information)

Scanning electron microscopy (SEM) analysis of well polished surfaces of pristine GeTe and Ge_0.92_Sb_0.08_Te crystals (Figure S4a,b, Supporting Information) clearly indicated the absence of precipitates or other impurities. Representative microscale images of energy‐dispersive x‐ray analysis (EDAX; Figures S4c–e, Supporting Information) also revealed the homogeneity of constituent elements, confirming the absence of impurities and clusters. Figure 1e shows a selected bright field transmission electron microscope (TEM) image of a Bridgman‐grown Ge_0.92_Sb_0.08_Te crystal taken along the $\left[1\bar{1}0\right]$ direction. A typical herringbone‐like structure with alternating dark and bright contrasts is observed in the image and can be attributed to dopant‐induced symmetry breaking in the GST crystal lattice.^[^ ^37^, ^38^ ^]^ Figure 1f displays the high‐resolution TEM (HRTEM) image of domains that exhibit many distributed streaks within the grains. A high magnification view of a defect‐free region is shown in Figure 1g, and its corresponding selected area electron diffraction (SAED) pattern along the zone axis of $\left[1\bar{1}0\right]$ is shown as an inset in Figure 1g, which could be indexed to the rhombohedral phase (*R3m*). Additionally, nanoscale Ge vacancies and stacking faults are evident in Figure 1h–j. The stacking faults may result from the unique stacking arrangement of atoms in the alloyed compound. It must be emphasized that these kinds of planar defects located within the grains are distinct from the normal grain boundary defects (which separate two crystals orientations) and the dislocations (that are considered as linear defects). Such diverse microstructures are expected to enhance multiple scattering of phonons, which in turn reduces the thermal conductivity of these samples, as discussed further below.

### 2.2 TE Properties of Pristine and Doped GeTe

**Figure 2:**
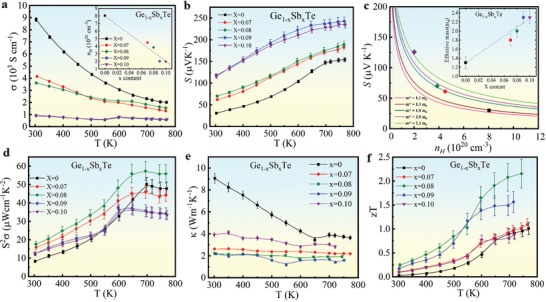
Thermoelectric properties of GeTe and Sb‐doped GeTe single crystals (GST). Temperature‐dependent a) electrical conductivity, the insert shows the room temperature experimental hole concentration *n_H_* as a function of Sb content (*x*), and b) Seebeck coefficient, c) Pisarenko plots in which the m* values were calculated within the single parabolic band model (smooth traces) by the fit of *n_H_* and Seebeck data, the insert shows the m* as a function of *x*. d) Temperature‐dependent *PF*, e) total thermal conductivity, and f) figure of merit.

The temperature‐dependent electrical conductivity *σ* of GST (Ge_1−_ *_x_*Sb*_x_*Te, *x* = 0–0.10) crystals in the range 300–800 K is shown in **Figure** **2**a. With increasing temperature the *σ* for GST crystals decreased for *x* = 0–0.08, which is indicative of the electrical conductivity behavior of a degenerate semiconductor. Specifically, with increasing Sb‐doping at the Ge sites (*x* > 0.08), a substantial reduction in *σ*, and a weak dependence on temperature are observed. As evident in Figure 2a, pristine GeTe exhibits a room temperature *σ* of ≈8.8 × 10^3^ S cm^−1^, which decreases by an order of magnitude to 0.9 × 10^3^ S cm^−1^ when x = 0.10 in GST. This significant reduction has been attributed to the Sb^3+^ ions either substituting the Ge^2+^ sites, or occupying the vacancy sites.^[^ ^27^ ^]^ The *n_H_* gradually decreased from 8.04 × 10^20^ cm^−3^ to 1.98 × 10^20^ cm^−3^ at 300 K as the concentration of Sb reached 10%. The reduction of carrier concentration *n_H_* with increasing Sb content was verified by the Hall measurement data (the inset in Figure 2a). Figure 2b displays the temperature dependence of the Seebeck coefficient (*S*) of the GST crystals. All samples used in this study exhibited a *p*‐type characteristic that increased with temperature and Sb concentration from 30 μVK^−1^ for *x* = 0 to 117 μVK^−1^ for *x* = 0.1 at 300 K (Figure 2b). In addition to the reduction in *n_H_*, a high density of states (DOS) effective mass (m*), which is favorable for the observed enhanced *S*, is shown in the Pisarenko plot^[^ ^39^ ^]^ of *n_H_* versus *S* for our GST crystals (Figure 2c). Furthermore, the m* values were calculated within the single parabolic band model (represented by smooth traces in Figure 2c) that was used to fit the room temperature experimental carrier concentration *n_H_* data (indicated by the data points in Figure 2c). The m* at room temperature increased from 1.3 *m*_o_ for pristine GeTe to 2.3* m*_o_ for *x* = 0.10 GST (the inset in Figure 2c). This gradual increase in m* and enhancement of *S* with increasing Sb‐doping has been attributed to a symmetry related band geneneracy arising from the tendency of Sb dopant to shift the crystal symmetry towards the cubic phase.^[^ ^27^, ^40^ ^]^

Figure 2d shows the *PF* of our GST crystals as a function of temperature. Compared to pristine GeTe, the *PF* for x = 0.08 was greatly enhanced over the whole temperature range and reached a maximum value of ≈56 μWcm^−1^ K^−2^ at ≈760 K. We thus conclude *x* = 0.08 as the optimal dopant concentration which balances the trade‐off between *σ* and S due to Sb doping. Figure 2e shows the temperature dependence of the total thermal conductivity *κ* of GST (*x* = 0 to 0.10) in the temperature range 300–800 K. The *κ* of pristine GeTe was ≈9 W m^−1^ K^−1^ at 300 K, which decreased with increasing temperature to a minimum value of ≈3.6 W m^−1^ K^−1^ near the phase transition temperature ≈670 K. In addition, *κ* was also substantially reduced with increasing Sb‐doping. For instance, GST with *x* = 0.08 exhibited a *κ* ≈2.1 W m^−1^ K^−1^ at 300 K and ≈1.9 W m^−1^ K^−1^ at 743 K, indicating a 75% and 54% reduction respectively, compared to that of pristine GeTe. This significant reduction in *κ* in the GST is attributed to a reduction in both *κ*_*lat*_ and *κ*_*e*_. The temperature‐dependent *κ*_*e*_ (Figure 5a) was calculated from *σ* using the Wiedemann‐Franz relation *κ*_*e*_ = *LσT*. *κ*_*e*_ drastically reduced from ≈6.2 Wm^−1^ K^−1^ (in *x* = 0) to ≈1.3 Wm^−1^ K^−1^ (in *x* = 0.08) at 300 K with Sb doping, in good agreement with the reduction in carrier concentration (Figure 2a). On the other hand, *κ*_*lat*_ (= *κ* − *κ*_*e*_) decreased by 76% from ≈1.1 Wm^−1^ K^−1^ in pristine GeTe to ≈0.46 Wm^−1^ K^−1^ in *x* = 0.08 GST at 740 K (Figure 5). This value is close to that of amorphous GeTe (≈0.39 W m^−1^ K^−1^),^[^ ^41^ ^]^ and we attribute this drastic reduction to mass fluctuation scattering (atomic mass, *M*_Sb_ = 121.8, *M*_Ge_ = 72.6 g mol^−1^), alloying effects and lattice anharmonicity caused by Sb doping. The *κ*, *κ*_*lat*_ and *κ*_*e*_ of the other Sb‐dopant concentrations are shown in the Figures S5, S6, Supporting Information. *κ*_*lat*_ in GeTe and GST show distinct temperature behavior and the corresponding phonon scattering mechanisms will be discussed in the next section. For example, the steep decrease in *κ*_*lat*_ in GeTe compared to a gradual decrease in *κ*_*lat*_ in GST (Figures 5, Figure S5a, Supporting Information) can be attributed not only to enhanced phonon scattering due to alloying, vacancies, and stacking faults, but also to the lattice anharmonicity, which will be discussed in detail in the light of Callaway's model.

The temperature dependence of *zT* for all GST crystals is shown in Figure 2f. The *zT* is enhanced in all GST crystals due to a simultaneous enhancement of *PF* and reduction of *κ* via Sb doping with the highest *zT* of 2.2 at 740 K in the optimally doped GST (*x* = 0.08). It is noteworthy that the 8% Sb‐doped GST exhibited a *zT* that is ≈ 15% higher than the *zT* = 1.85 reported in Ref.^[^ ^27^ ^]^ for GST crystalline ingots. We attribute the high *zT* in our 8% Sb‐doped GST crystal due to a combined effect of enhanced TE *PF*, reduced thermal conductivity due to mass fluctuation scattering and alloying effects, and lattice anharmonicity caused by Sb doping. Lastly, we confirmed the reversibility of TE properties of the GST crystals after high temperature measurements (Figure S7, Supporting Information).

### 2.3 Origin of High *zT*

**Figure 3:**
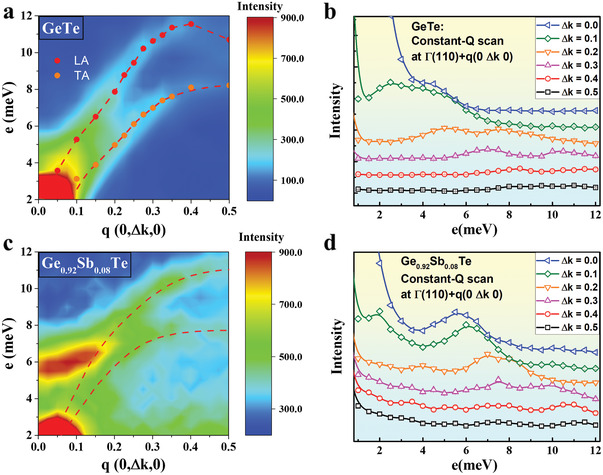
Inelastic neutron scattering studies of GeTe and Sb‐doped GeTe. Phonon dispersions relation from *S (Q, E)* with function of energy transfer *E* and *q* along [0K0] for a) pristine GeTe and c) Ge_0.92_Sb_0.08_Te crystals with TA and LA branches. The solid circles in (a) were determined by the multi‐peak Gaussian function from panel (b), and the red dashed lines are guides to the eye. (b) and (d) show phonon energy spectra for energy scans along [0K0] with a constant *Q* of *k* = 1–1.5 for GeTe and Ge_0.92_Sb_0.08_Te crystals respectively. The open symbols represent the data collected from the triple‐axis spectrometer of SIKA, while the solid lines in (b) are numerical fits with a multi‐peak Gaussian function, and in (d) are guides to the eye.

In recent years, high *zT* has been achieved in TE materials via nanostructuring approaches that enable prominent reduction of *κ*_*lat*_ via modification of their acoustic phonon spectrum. Although such phonon engineering approaches are generally associated with nanostructured materials, Kargar et al.^[^ ^42^ ^]^ reported a dopant‐induced modification of the phonon dispersion that could have important implications for TE materials, as well enable development of thermal management materials and optoelectronic devices. In this regard, we focus on the implications of Sb‐doping on the phonon structure of GST in order to understand the origin of the high *zT*. We first discuss the phonon dispersions of pristine and doped GST (x = 0.08) elicited from our INS measurements. The scattering function *S(* ***Q*** *,E)* is presented on the basis of INS measurements (conducted at on a cold triple axis spectrometer, Sika, ANSTO) on GST crystals, and the results of ab initio phonon calculations using DFT. **Figure** **3** shows the INS results from GeTe and Ge_0.92_Sb_0.08_Te, and highlights the greater complexity of the phonon dispersion of Ge_0.92_Sb_0.08_Te compared to that of GeTe. Figure 3a presents the *S(* ***Q*** *,E)* map and energy profile for pristine GeTe at selected *q* vectors with steps of 0.05. A multi‐peak Gaussian function was employed to fit each experimental energy profile and to extract the energies of the transverse acoustic (TA) and longitudinal acoustic (LA) phonons at each *q*. These energies are plotted as the solid circles in Figure 3a and represent the TA and LA branches in the phonon dispersion. The corresponding data from GST are significantly different. An unexpected extra excitation between 5–7 meV is observed for the Ge_0.92_Sb_0.08_Te crystal (Figure 3c). In addition, the energy profile (Figure 3d) of Ge_0.92_Sb_0.08_Te exhibits a higher complexity compared to that of pristine GeTe, making it challenging to extract peak energies by fitting the profiles to multiple Gaussian peaks. Instead, we plot dashed lines in Figure 3c as guides to the eye, tracing the probable TA and LA phonon branches, which exhibit lower slopes compared to pristine GeTe.

**Figure 4:**
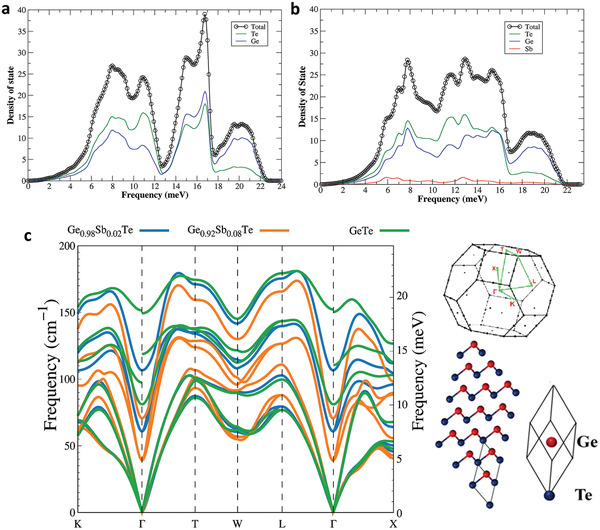
Phonon density of states and dispersion relations. Theoretical calculation of partial density of states for a) GeTe and b) Ge_0.92_Sb_0.08_Te crystals. The red trace in (b) indicates the contribution from Sb. c) The VCA method based on first‐principles calculations for examining of the effects of partially doping Sb into GeTe on phonon dispersion and ‐PDOS. Two Sb doping levels 0.02 (blue traces) and 0.08 (orange traces) are shown for comparing to that of pristine GeTe (green traces). The right panel shows the supercell used in the DFT calculation of GeTe. Similar DFT calculations were performed for GST with different Sb‐concentrations, and the overall phonon frequency softens due to the Sb doping. In pristine GeTe, no phonon modes are present between ≈0 and 10 meV at the *Γ* point and 12–13 at *W* point.

To gain further insight, we calculated the partial phonon density of states (PDOS) (**Figure** **4**a,b), which also exhibit additional features between ≈5–7 and ≈12–13 meV for GST. We attribute these features to the presence of Sb dopants. Furthermore, we applied DFT using the virtual crystal approximation (VCA) method to calculate the phonon dispersion relations of pristine GeTe and GST (x = 0.08), which are shown in Figure 4c. The presence of a phonon around 5 meV at the *Γ* point can clearly be seen in the dispersion of Ge_0.92_Sb_0.08_Te (orange traces), which is not present in pristine GeTe (green traces). This corroborates our observation of the extra excitation in the *S(* ***Q*** *,E)* map of GST (Figure 3b). The calculated phonon dispersions also reveal an overall softening of the phonon frequencies in Ge_0.92_Sb_0.08_Te owing to the Sb doping. This softening can also be observed in GST with a lower Sb concentration (Ge_0.98_Sb_0.02_Te, blue traces), where an intermediate phonon mode is present at ≈5–7 meV at the *Γ* point. In contrast, no phonon modes are present between ≈0–10 meV at the *Γ* point in pristine GeTe. The additional phonons in GST could provide extra decay channels for optical phonons, thereby increasing their scattering rate and lowering the thermal conductivity.

**Figure 5:**
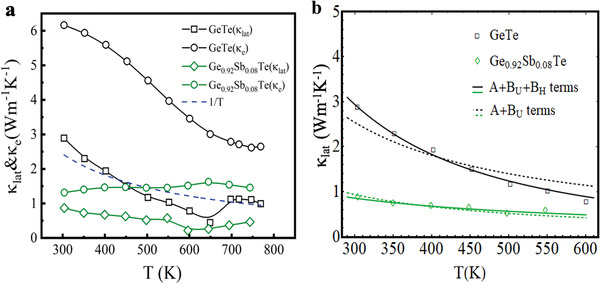
Anharmonicity in Sb‐doped GeTe. a) Electronic (*κ*_e_) and lattice (*κ*_lat_) contributions to thermal conductivity as a function of temperature for pristine GeTe and doped GST (*x* = 0.08) single crystals. The dotted blue line shows the 1*/T* dependence. b) The dotted black line shows the *1/T* dependence that results from Umklapp scattering (three‐phonon), which is inadequate to describe the temperature behavior exhibited by pristine GeTe. The solid black line includes an additional four‐phonon scattering process in pristine GeTe that can describe the temperature dependence of the measured *κ*_lat_ more accurately. In addition to point defect scattering, three phonon scattering is the more prominent scattering mechanism in GST (*x* = 0.08) single crystal (solid green line).

Returning to the lattice thermal conductivity, we note that the *κ*_*lat*_ of pristine GeTe exhibts a temperature dependence that is distinct from the expected 1*/T* behavior at high temperatures arising from (three‐phonon) Umklapp scattering (**Figure** **5**a). As suggested by Slack and Glassbrenner,^[^ ^43^ ^]^ *κ*_*lat*_ at high temperatures (*θ* > *θ*_*D*_) can be described by a modified Callaway's model^[^ ^44^ ^]^ as

(1) $$\begin{array}{c} \kappa_{lat}=\frac{k_{B}}{2\pi^{2}v_{s}}{\left(\frac{k_{B}T}{\hbar }\right)}^{3}\int_{0}^{\theta_{D}/T}\tau_{c}x^{2}dx \end{array}$$

where *x* = ℏ*ω*/*k_B_T* is the reduced phonon frequency, *ω* the phonon frequency, *v_s_* the velocity of sound, *k_B_* the Boltzmann constant and ${\tau_{C}}^{-1}=\sum_{i}\tau_{i}^{-1}={\tau_{PD}}^{-1}+{\tau_{U}}^{-1}+{\tau_{H}}^{-1}+{\tau_{B}}^{-1}+\cdot \cdot \cdot =A\omega^{4}+\left(B_{U}Te^{-\theta_{D}/3T}+B_{H}T^{2}\right)\omega^{2}+\frac{v_{s}}{L}+\cdot \cdot \cdot$ is the combined phonon relaxation time, assuming that the phonon scattering effects are additive following Matthiessen's rule. Here, *A* is the point defect scattering parameter, *B_U_* and *B_H_* are the scattering parameters for the Umklapp (three‐phonon) and higher order four‐phonon scattering processes, and *v*_s_ */L* represents the boundary scattering (dominant at low temperatures), respectively. As the name suggests, a three‐phonon process involves the lowest order anharmonic coupling resulting in the decay of a single phonon into two phonons or the recombination of two phonons into a new phonon, while the four‐phonon process involves four phonon interactions through either recombination or decay. To analyze the high temperature dependence of *κ*_*lat*_ in pristine and Sb‐doped GeTe, we ignore the contributions from boundary scattering in the temperature range (300–750 K) and simplify Equation (1) as

(2) $$\begin{array}{ccc} \kappa_{lat} & \approx & \frac{k_{B}}{2\pi^{2}v_{s}}{\left(\frac{k_{B}T}{\hbar }\right)}^{3}\int_{0}^{\theta_{D}/T}\frac{x^{2}}{A\omega^{4}+\left(B_{U}Te^{-\theta_{D}/3T}+B_{H}T^{2}\right)\omega^{2}}\\ dx & = & \frac{\hbar }{2\pi^{2}v_{s}ATx_{0}\left(T\right)}\tan^{-1}\left[\frac{\theta_{D}}{Tx_{0}\left(T\right)}\right] \end{array}$$

where $x_{0}\left(T\right)=\sqrt{\frac{B_{U}Te^{-\theta_{D}/3T}+B_{H}T^{2}}{A{\left(\frac{k_{B}T}{\hbar }\right)}^{2}}}$. The intermediate steps and detailed fitting results are given in the SI section. As shown in Figure 5b, clearly only the three‐phonon Umklapp scattering along with point defect scattering (shown as the black dashed trace) is inadequate to describe the temperature dependence of *κ*_*lat*_ exhibited by pristine GeTe and hence, inclusion of the four‐phonon scattering process is necessary to accurately describe its temperature dependence. This has been observed previously in crystals which exhibit phonon gaps, that is, a gap in the phonon dispersion between the acoustic and optical phonon energies. Such a gap limits the number of decay channels for optical phonons, necessitating a four‐phonon decay.^[^ ^45^ ^]^ On the other hand, a three‐phonon process is sufficient to describe the temperature dependence of *κ*_*lat*_ for Ge_0.92_Sb_0.08_Te. This temperature dependence further supports our earlier observation (from Figure 4) of the availability of a higher number of decay channels, resulting in high phonon scattering rates. Moreover, Ge vacancies also act as phonon scattering centers in Ge_1−_ *_x_*Sb*_x_*Te (*x* = 0, 0.08) and the scattering parameter for point defect scattering estimated by Klemens represents a combination of vacancy^[^ ^46^ ^]^ and mass fluctuation scattering^[^ ^47^ ^]^ terms as given by

(3) $$\begin{array}{c} A\approx \frac{V}{4\pi v_{s}^{3}}\left[N_{V}{\left(-\frac{M_{a}}{M}-2\right)}^{2}+x\left(1-x\right){\left(\frac{M_{Ge}-M_{Sb}}{M^{\prime }}\right)}^{2}\right] \end{array}$$

where *V* is the volume per atom, *v_s_* is the phonon velocity (or the velocity of sound through the material), *N_V_* is the relative vacancy concentration, *M* is the average mass per atom, *M_a_* is the mass of the missing atom, the term ‐2 accounts for the potential energy of the missing linkages, or twice the potential energy per atom,^[^ ^46^ ^]^ *M*_Ge_ = 72.63 g mol^−1^, *M*_Sb_ = 121.76 g mol^−1^, and *M′* is the average mass per ternary cluster Ge_1−_ *_x_*Sb*_x_*Te. **Table** **1** shows the fitting parameters and the calculated theoretical values of *A* and *B* (where, $B_{U}\approx \frac{\hbar \gamma^{2}}{Mv_{s}^{2}\theta_{D}})$ ^[^ ^48^ ^]^ that provides a theoretical validation of our fit. Surprisingly, both fitted and calculated values of *A* for pristine GeTe are comparable to that of the Sb‐doped GeTe, which is counterintuitive, since the second term in Equation (3) due to mass fluctuation scattering by an impurity atom is absent in pristine GeTe. Nonetheless, the phonon scattering strength by vacancies is much stronger than that of mass fluctuation scattering due to Sb^3+^ ions substituting at Ge^2+^ sites, since vacancy scattering is linked to both scattering of phonons by missing mass and missing interatomic linkages.^[^ ^46^ ^]^ Here, the scattering strength by vacancies in Ge_0.92_Sb_0.08_Te is further compensated by mass fluctuation scattering (*M_Ge_* − *M_Sb_*), as many of the vacancies are now occupied by Sb^3+^ ions in addition to substituion at the Ge^2+^ sites, which also led to the enhancement of *S* due Sb‐doping (*cf*. Figure 2b). From Table 1, we see that the calculated value of *A* (*B_U_*) differ by an order (roughly two orders) of magnitude from our fitted values that is attributed to the constant *v_s_* in the Callaway's model which assumes a single averaged phonon velocity approximately equal to the velocity of sound without any distinction between longitudinal and transverse velocities.^[^ ^33^, ^42^ ^]^ Notably, in GST the phonon dispersion is distinctly different from that of pristine GeTe leading to their different fitted *B*_U_ values (Table 1). Although from Figure 5b, it is evident that the point defect and three‐phonon scattering processes are adequate to model the temperature dependent behavior of *κ*_*lat*_ in GST, based on the presence of stacking faults and herringbone structures in Figure 1e, we also considered an additional phonon scattering via stacking faults ($\tau_{SF}^{-1}=0.7\frac{a_{lat}^{2}\gamma^{2}N_{s}\omega^{2}}{v_{s}}$),^[^ ^49^ ^]^ where *a_lat_* is the average lattice constant, and *N_s_* is the number of stacking faults per meter and the results are shown in Table S1, Supporting Information.

**Table 1:** Fitting parameters used in the Callaway's model, where *A* is the point defect scattering parameter, and *B*_U_ and *B*_H_ are three‐ and four‐phonon Umklapp scattering parameters, respectively

Scattering parameters	GeTe		Ge_0.92_Sb_0.08_Te	
	Fitted (solid black, Figure 5b)	Calculated	Fitted (solid green, Figure 5b)	Calculated
*A* (s^3^)	5.03 × 10^−42^	4.90 × 10^−41^	4.33 × 10^−41^	2.57 × 10^−41^
*B*_U_ (sK^−1^)	8.97 × 10^−21^	2.81 × 10^−18^	2.24 × 10^−17^	2.76 × 10^−18^
*B*_H_ (sK^−2^)	2.48 × 10^−20^	–	≈0	–

As an additional confirmation of the data shown in Figure 5, we analyzed the temperature‐dependent polarized Raman data of GeTe reported in Ref.^[^ ^34^ ^]^ Similar to the case for the *κ*_*lat*_, we found that the phonon decay in GeTe is dominated by a four‐phonon process (*cf*., Figure S8, Supporting Information). As shown in the phonon dispersions in Figure 4c, Sb‐doping results in a softening of the phonon energies and the emergence of new phonon mode around 5 meV (≈40 cm^−1^), which could be observed in the Raman spectrum of GST. However, considering the difficulties in sample preparation for polarized Raman scattering studies, the temperature‐induced broadening and low intensity of a peak close to the laser line (at 0 cm^−1^ shift), we believe our INS measurements, calculated DOS and dispersions, unambiguously highlight the differences in the phonon structure of pristine GeTe and GST.

## 3 Conclusion

Bulk GeTe exhibits a comparatively high total thermal conductivity *κ* but a surprisingly low lattice thermal conductivity *κ*_*lat*_ at high temperatures, which is further reduced via Sb‐doping through a simultaneous reduction in *κ*_*e*_ and *κ*_*lat*_. Doping by Sb also results in an enhancement in the Seebeck coefficient, which is the key to achieving a high *zT* of 2.2 at 740 K in Ge_0.92_Sb_0.08_Te. Here we uncovered the origin of the significantly low *κ*_*lat*_ of Sb‐doped GeTe experimentally and theoretically. Our INS measurements revealed significant differences in the phonon dispersion, and the presence of a new phonon band at a transfer energy *E* ≈ 5–6 meV in Ge_0.92_Sb_0.08_Te. The phonon dispersion and density of states calculations also revealed the presence of additional states owing to the Sb dopant, which also softened the phonon frequencies in GST. These effects combine to lower the *κ*_*lat*_ in GST, which approaches that of amorphous GeTe at high temperatures. From the temperature dependence of *κ*_*lat*_ and analysis of Raman spectra of GeTe, we infer that a three‐phonon Umklapp scattering process is the dominant scattering mechanism in GST, in contrast to pristine GeTe where a four‐phonon process is the dominant mechanism. Our measured low *κ* in GST is attributed to this dopant related change in the phonon spectra, which provides a new direction in phonon engineering of single crystalline TE materials beyond nanostructuring.

## 4 Experimental Section

##### Synthesis—Starting Materials

Ge ingot (99.999%), Sb shot (99.999%), and Te shot (99.999%) were purchased from Alfa Aesar. All elements were further purified several times before use.

##### Synthesis—Bridgman Crystal Growth

We followed a two‐step strategy for obtaining high‐quality Ge_1−_ *_x_*Sb*_x_*Te, GST (hereinafter) (*x* = 0–0.10) single crystal. First, to ensure homogeneous mixing of compounds, bulk ingots (≈20 g) of GST (*x* = 0, 0.07, 0.08, 0.09, and 0.10) were synthesized by weighing stoichiometric ratio of purified elements Ge, Sb, and Te into quartz tubes. The quartz tubes were evacuated to ≈10^−6^ torr, and slowly heated to 1223 K for 6 h and soaked at this temperature for 48 h and followed by furnace cooling to room temperature. Next, the as‐synthesized ingots were crushed into small pieces by using a mortar and pestle and sealed in quartz tubes (≈10 × 12 × 300 mm^3^) with pointed conical ends under a high vacuum (≈10^−6 ^torr). The conical tube was then placed inside another bigger quartz tube (≈13 × 16 × 300 mm^3^) to prevent any cracking caused by thermal expansion. The GST single crystals ≈ 12 mm in diameter and ≈20 g in weight were then grown by using vertical Bridgman method from the pre‐melt ingots, with a growth rate of 3 mm hr^−1^ (*cf*. Figure 1a). The samples were then cut into desired diameters using a diamond wheel saw for TE characterization.

##### Characterization—Structure and Crystallinity Characterization

The orientation of *a*, *b*, and *c* axes were determined by Laue diffractometer. A schematic of crystal and lattice axes of GeTe crystal is shown in Figure S1, Supporting Information that indicates the direction along which the as‐grown crystals were cut for electronic and thermal transport measurement. The XRD patterns of GST crystals were collected by using PANalytical X'Pert PRO X‐ray diffractometer with a Cu K*α* X‐ray source (*λ* = 1.5406 Å). The lattice parameters of crystals were determined by using Rietveld refinement from the Highscore Plus program. The structural analysis of as grown single crystal samples are measured by using Panalytical X‐ Pert Pro MRD X‐ray diffractometer (Pw3040/60).

##### Characterization—Electrical Resistivity and Seebeck Coefficient

The longer direction coincides with the direction in which thermal conductivity is measured. Electrical resistivity and Seebeck coefficient of the GST crystals were measured simultaneously on 2 × 2 × 8mm^3^ parallelepiped bar samples using a commercially available instrument (ZEM‐3, ULVAC‐RIKO, Japan) from 300–800 K under He‐gas atmosphere. The uncertainty of the Seebeck coefficient and electrical conductivity measurements is about ≈2%–4% and is about 10% for the *PF*.

##### Characterization—Thermal Conductivity

The thermal conductivity *κ* (= *DρC_p_*) of single crystalline GST (≈10 mm in diameter and ≈1.6 mm in thickness) from 300–773 K were calculated by measuring their thermal diffusivity *D* using the laser flash instrument (NETZSCH, LFA457, Cowan model with pulse correction), the density *ρ* (using traditional Archimedes method), and temperature‐dependent heat capacity *C_p_* using DSC Q‐100 for 300–673 K and Quantum Design Physical Property Measurement System for 2–350 K (Figure S1, Supporting Information).The repeatability of measurement undergone thermal cycling confirmed a good thermal stability of the crystals.

##### Characterization—Hall Measurement

The room temperature Hall coefficient *R*_H_of the GST crystals were measured using a commercial Quantum Design Physical Property Measurement System (PPMS) via scanning a magnetic field from −2T to +2T and an applied current of 5 mA. The carrier concentration was estimated using *n_H_* = − 1/*eR_H_*, where *e* is the electronic charge. The uncertainty of the Hall coefficient is ≈3%.

##### Characterization—Electron Microscopy of SEM and TEM

The surface morphology and elemental composition analysis of the GST single crystals were probed using the SEM (Inspect F FEI) equipped with energy dispersive x‐ray spectroscopy (EDX). The GST sample with 70–80 nm in thickness was cut from its bulk ingot using a Focused Ion Beam instrument (Hitachi NX2000). The TEM micrographs and SAED patterns were acquired using a TEM (JEOL JEM‐2100) at an accelerating voltage of 200 kV.

##### Characterization—Inelastic Neutron Scattering Measurement

The INS technique allows us to discover the phonon dispersion relation by mapping of scattering function, *S(* ***Q*** *,E)* for studying the microscopic dynamics of materials. In this work, the beam line SIKA—the multiplexing cold‐neutron triple‐axis spectrometer at ANSTO (Australia) is employed to perform the energy scan of *q* along [0K0] for mapping of *S(* ***Q*** *,E)*, where *E* is the energy transfer and ***Q = Γ + q*** the wave‐vector transfer, with *q* a wave vector and ***Γ*** a reciprocal lattice. The single crystals of mass *m* ≈ 20–25 g were cut from chunks that were grown by the Bridgman method. In this work, energy scan of constant‐*q* along [0k0] are employed to map the *S(* ***Q*** *,E)* as a function of energy transfer *E* and wave‐vector transfer ***Q*** *= Γ + q*, from *Γ* at (110) to the zone boundary in the single Brillouin zone with final energy *E_f_* = 14.87 mV, where the scan step of *E* and *q* are 0.5 meV and 0.05, respectively.

##### First‐Principle Calculations

The first‐principle DFT was performed using projector augmented‐wave (PAW) potentials^[^ ^50^ ^]^ as implemented in the Vienna ab initio simulation package (VASP).^[^ ^51^, ^52^, ^53^ ^]^ The exchange‐correlation function was treated by the generalized gradient approximation with the Perdew–Burke–Ernzerhof form.^[^ ^54^ ^]^ The experimental lattice constants with their atomic positions fully relaxed were used for the pristine and Sb doped GeTe calculation. In order to study the Sb doping effects of GeTe, we used both VCA within DFT scheme implemented in Quantum ESPRESSO^[^ ^55^, ^56^ ^]^ and rigorous DFT calculations (using VASP) combined with phonopy.^[^ ^57^ ^]^ VCA is a simple method to study the disordered alloys and solid solutions, in which the primitive unit cell but with spurious’ virtual’ atomic potential interpolating between the host and doping atoms is applied. Even though this approach neglects the local deformations in the vicinity of atoms and less accurate in exploring the disordered structures, it usually yields useful and reasonable results. In this work, the Sb doping is introduced by the virtual pseudopotential of Ge_1−_ *_x_*Sb*_x_* that composed from the actual Ge and Sb pseudopotentials: *V*_VCA_ = *xV*_Ge_ + (1 − *x*)*V*_Sb_ where *V*_Ge_ and *V*_Sb_ are pseudopotential of Ge and Sb respectively. The norm‐conserving pseudopotentials of Hartwigsen–Goedecker–Hutter and an energy cutoff of 70 Ry were used along with a Monkhorst–Pack *k*‐point mesh of 12 × 12 × 12 for Brillouin Zone sampling in electronic structure calculations. The VCA phonon dispersion was performed in the framework of density functional perturbation theory (DFPT) using a 4 × 4 × 4 q mesh. The PDOS ‐was calculated using the phonopy code with force constants obtained from rigorous VASP calculations.

##### Quantifying Anharmonicity through Raman Spectroscopy

We used the temperature‐dependent polarized Raman spectroscopy data with *z(xx)z* and *z(xy)z* configurations from Ref [34] and modeled the temperature dependent shifts in the frequency of both *E* and *A* modes,^[^ ^58^, ^59^, ^60^, ^61^, ^62^, ^63^ ^]^ as described in the SI section.

## Conflict of Interest

The authors declare no conflict of interest.

## Supporting information

Supporting Information
