# Supplementary material for: High zT and Its Origin in Sb‐doped GeTe Single Crystals
Source: Adv Sci (Weinh). 2020 Nov 6;7(24):2002494. doi: 10.1002/advs.202002494 (PMC7740100; doi:10.1002/advs.202002494)
Supplement: Supplementary file 1 — Supporting Information [file ADVS-7-2002494-s001.pdf]

© 2020 Wiley-VCH GmbH

ADVANCED SCIENCE
Open Access

Supporting Information

for *Adv. Sci.*, DOI: 10.1002/advs.202002494

High *zT* and its origin in Sb-doped GeTe single crystals

Ranganayakulu K.Vankayala, Tian-Wey Lan, Prakash Parajuli, Fengjiao Liu, Rahul Rao, Shih Hsun Yu, Tsu-Lien Hung, Chih-Hao Lee, Shin-Ichiro Yano, Cheng-Rong Hsing, Duc-Long Nguyen, Cheng-Lung Chen, Sriparna Bhattacharya,* Kuei-Hsien Chen, Min-Nan Ou, Oliver Rancu, Apparao M. Rao, and Yang-Yuan Chen**

Supporting Information

High *zT* and its origin in Sb-doped GeTe single crystals

Ranganayakulu K.Vankayala, Tian-Wey Lan, Prakash Parajuli, Fengjiao Liu, Rahul Rao, Shih Hsun Yu, Tsu-Lien Hung, Chih-Hao Lee, Shin-Ichiro Yano, Cheng-Rong Hsing, Duc-Long Nguyen, Cheng-Lung Chen, Sriparna Bhattacharya,* Kuei-Hsien Chen, Min-Nan Ou, Oliver Rancu, Apparao M. Rao, Yang-Yuan Chen,**

R. K. Vankayala, Dr. T-W Lan, Dr. S. H. Yu, Dr. T-L Hung, Dr. C-L Chen,* Dr. M-N Ou, Prof. Y-Y. Chen*
Institute of Physics, Academia Sinica, Taipei 11529, Taiwan, ROC
Email: aabbss@gate.sinica.edu.tw, cheny2@phys.sinica.edu.tw
R. K. Vankayala, Prof. C-H Lee
Dept. of Engineering and System Science, National Tsing Hua University, Hsinchu 30013, Taiwan, ROC
R. K. Vankayala
Taiwan International Graduate Program, Taipei 115, Taiwan, ROC
P. Parajuli, F. Liu, Dr. S. Bhattacharya,* Oliver Rancu, Prof. A. M. Rao
Clemson Nanomaterials Institute, Department of Physics & Astronomy, Clemson University, Clemson, South Carolina 29634, USA
Email: bbhatta@g.clemson.edu
Dr. Rahul Rao
Air Force Research Laboratory, WPAFB, Dayton, Ohio 45433, USA
Dr. S-I Yano
National Synchrotron Radiation Research Center, Hsinchu 30077, Taiwan, ROC
Dr. C-R Hsing, Dr. D-L Nguyen, Prof. K-H. Chen
Institute of Atomic and Molecular Sciences, Academia Sinica, Taipei 10617, Taiwan, ROC

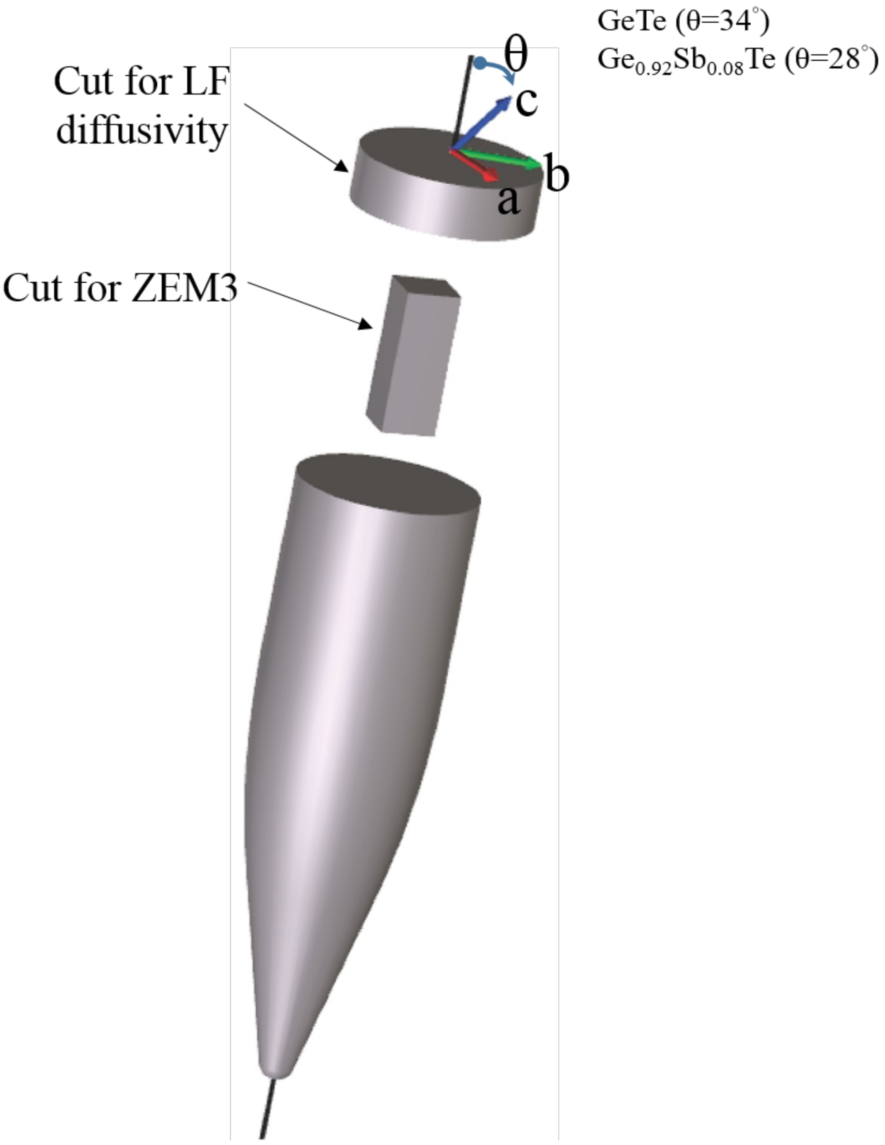

Fig. S1. A schematic showing the orientation of the cut samples used in this study. Here, *a*, *b*, *c* and θ represents respectively the crystallographic axes and the tilt angle with respect to the normal.

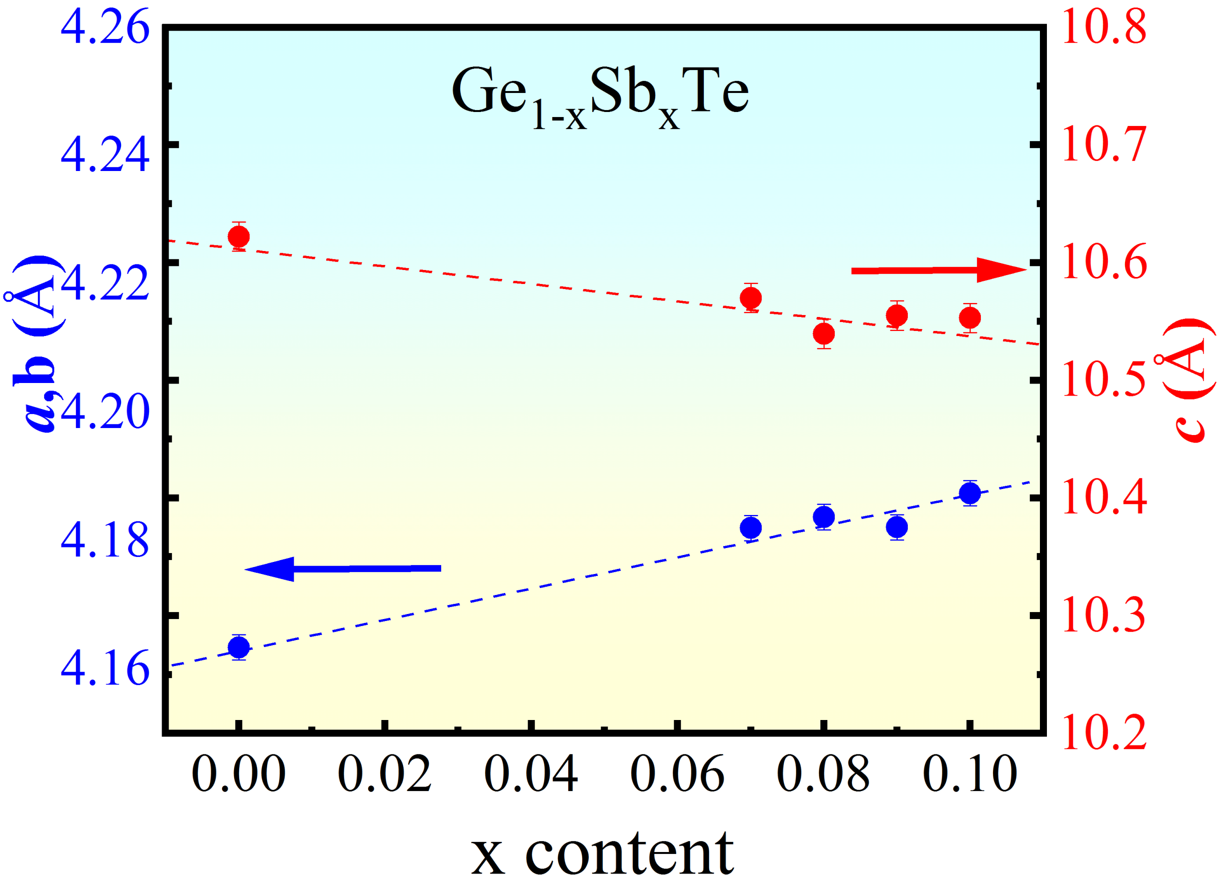

Fig. S2. Composition *x* dependent lattice parameters of GST deduced from the Rietveld refinement.

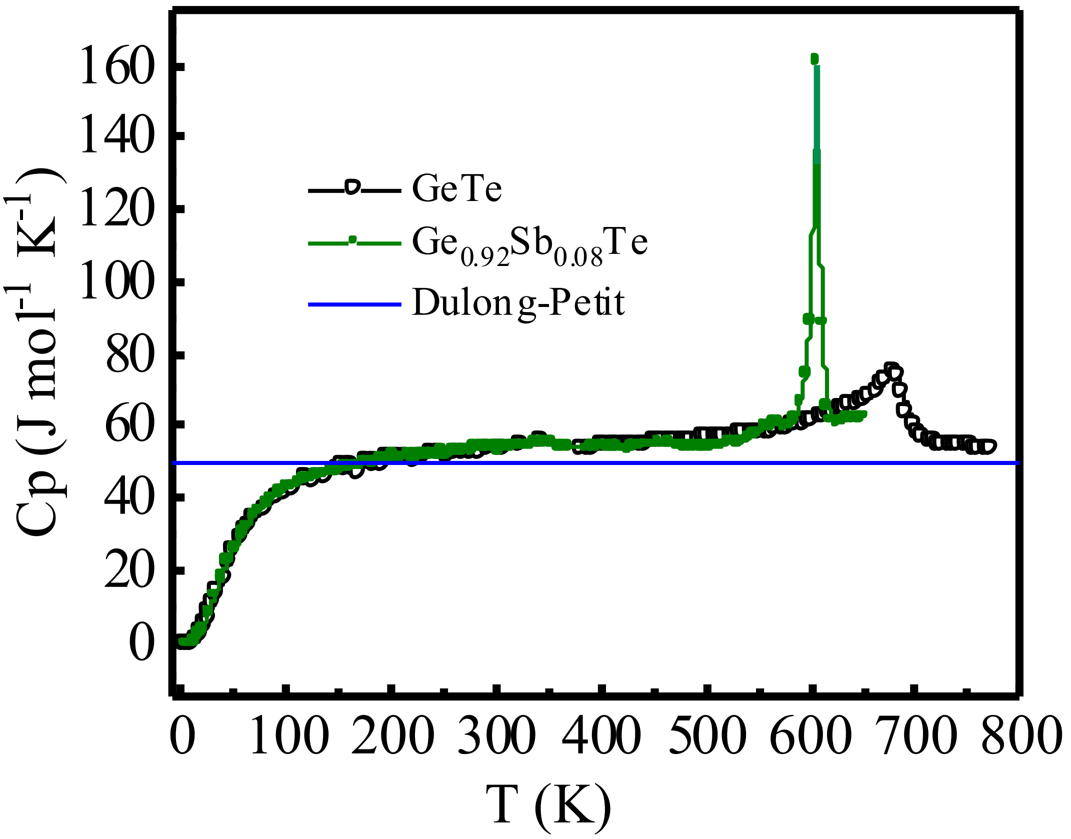

Figure S3: Specific heat capacity of GeTe and $Ge_{0.92}Sb_{0.08}Te$ as a function of temperature measured using the PPMS (low temperature) and the DSC Q-100 (high temperature). A peak shift in GST indicates the shift of T_c towards a lower temperature with Sb-doping.

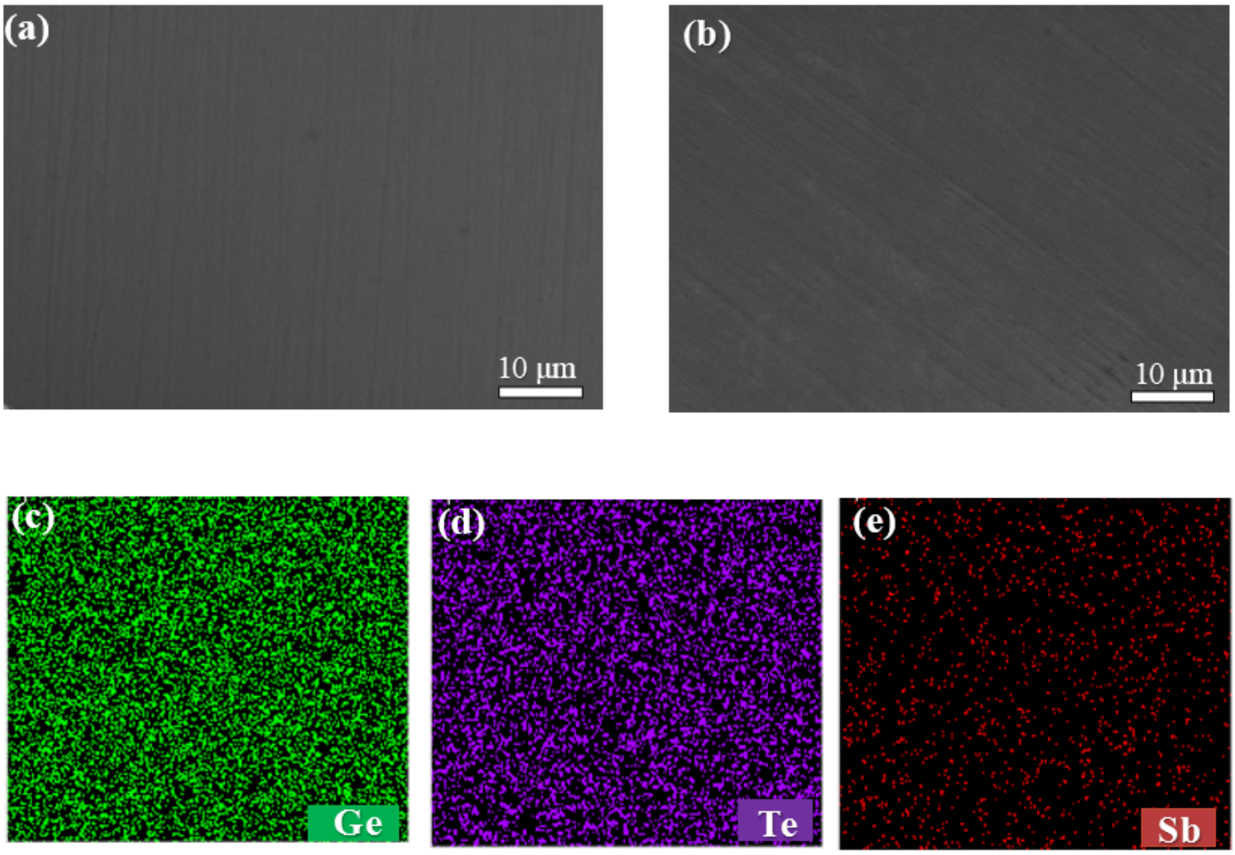

Figure S4: (a) & (b) SEM images of carefully polished surface samples of GeTe and $Ge_{0.98}Sb_{0.08}Te$. Few scratches due to polishing are evident. Panels (c) (d) & (e) represent the elemental mappings for $Ge_{0.98}Sb_{0.08}Te$ obtained using EDAX.

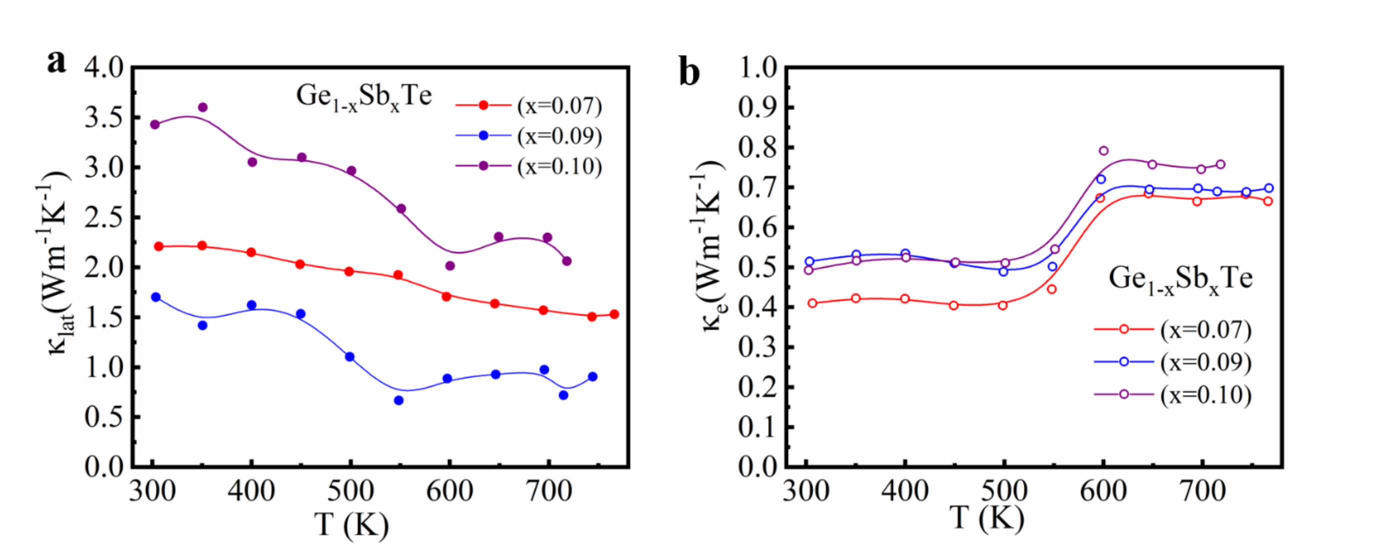

Figure S5: Lattice and electronic contribution to thermal conductivity of GST (x = 0.07, 0.09, 0.10) crystals.

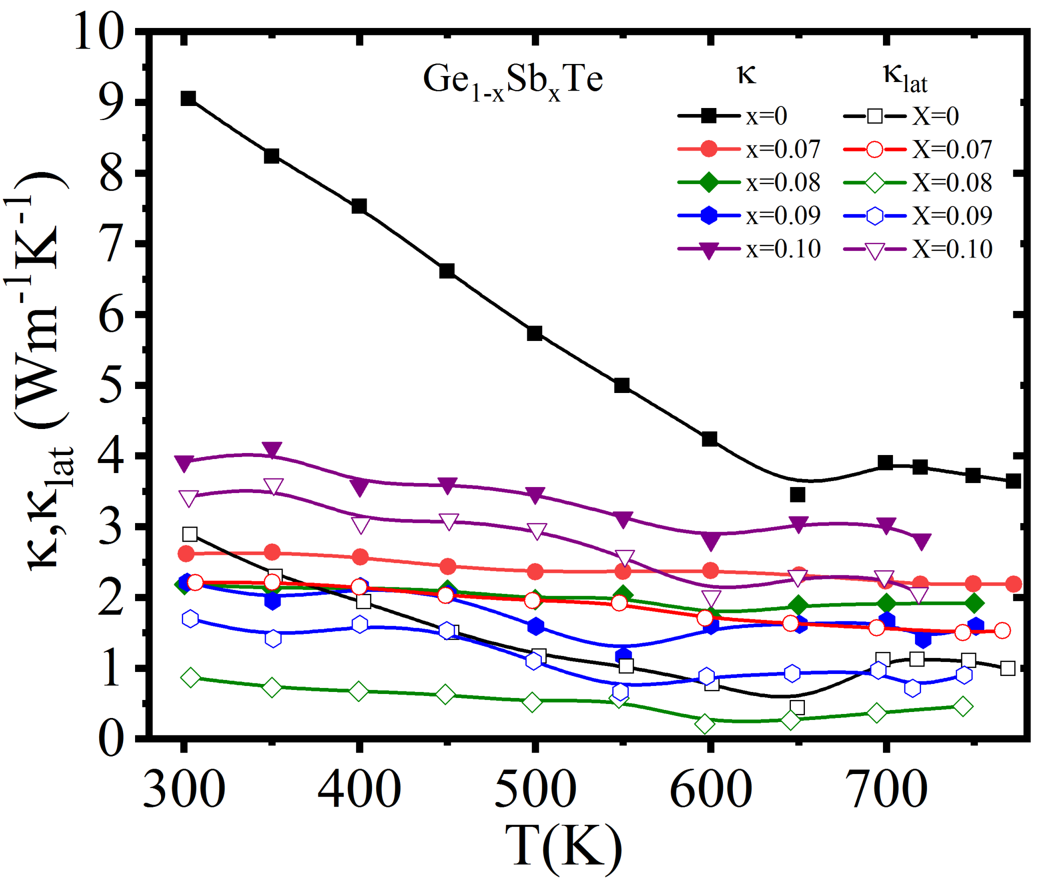

Figure S6: Total and lattice contribution to thermal conductivity of GST (x = 0, 0.07, 0.08, 0.09, 0.10) crystals.

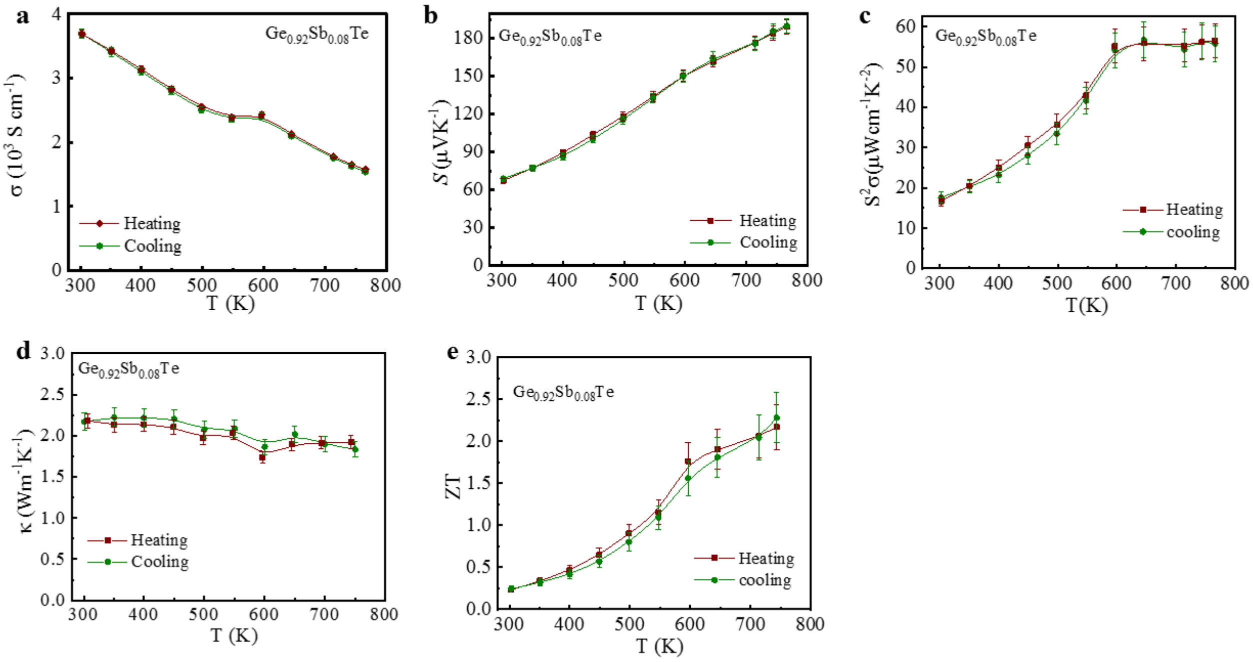

Figure S7: The reversibility of thermoelectric properties of $Ge_{0.92}Sb_{0.08}Te$. The heating and cooling data match well and are within the error bar region.

Analysis of temperature dependent lattice thermal conductivity of $Ge_{1-x}Sb_xTe$ (x=0, 0.08)

Simplification of Callaway's model (eqn. 1):

$$\kappa_{lat} = \frac{k_B}{2\pi^2 v_s}\left(\frac{k_B T}{\hbar}\right)^3 \int_0^{\theta_D/T} \tau_c x^2 dx \quad (1)$$

$$\kappa_{lat} \approx \frac{k_B}{2\pi^2 v_s}\left(\frac{k_B T}{\hbar}\right)^3 \int_0^{\theta_D/T} \frac{x^2}{A\omega^4 + (B_U T e^{-\theta_D/3T} + B_H T^2)\omega^2} dx$$

$$\kappa_{lat} = \frac{k_B}{2\pi^2 v_s}\left(\frac{k_B T}{\hbar}\right)^3 \int_0^{\theta_D/T} \frac{x^2}{A\left(\frac{k_B T x}{\hbar}\right)^4 + (B_U T e^{-\theta_D/3T} + B_H T^2)\left(\frac{k_B T x}{\hbar}\right)^2} dx$$

$$\kappa_{lat} = \frac{\hbar}{2\pi^2 v_s A T} \int_0^{\theta_D/T} \frac{1}{x^2 + [x_0(T)]^2} dx$$

Where, $x_0(T) = \sqrt{\frac{B_U T e^{-\theta_D/3T} + B_H T^2}{A\left(\frac{k_B T}{\hbar}\right)^2}}$

Solving above equation we get,

$$\kappa_{lat} = \frac{\hbar}{2\pi^2 v_s A T x_0(T)} \tan^{-1}\left[\frac{\theta_D}{T x_0(T)}\right]. \quad (2)$$

To show the necessity of including a four phonon scattering process to describe the steeper fall of temperature dependent thermal conductivity of GeTe, initially only point defect scattering and three phonon Umklapp scattering were considered within equation (2) by deliberately setting $B_H = 0$. The resulting fit (as represented by the dashed trace in Fig. 5b) does not explain the experimental results. The inclusion of a four phonon scattering process yielded a much better fit as shown in Fig. 5b by the solid black trace. A similar approach was employed to describe the temperature profile of Sb doped GeTe data below the phase transition temperature, and both fits with (solid green trace) and without (dashed green trace) the 4-phonon scattering are in close agreement as shown in Fig. 5b.

A) Calculated Parameters:

1) Pristine GeTe,

Only Ge vacancies are considered while calculating point defects,

$$\tau_{PD}{}^{-1} = \frac{V}{4\pi v_s^3}\Gamma\omega^4$$

where,

$$\Gamma = \Gamma_{MF} + \Gamma_{Strain}$$
$$= \Gamma_{MF}(vacancy) + \Gamma_{MF}(doped) + \Gamma_{Strain}$$
$$= N_V\left(\frac{\Delta M}{M}\right)^2 + \sum_i x_i\left[\left(\frac{\Delta M_i}{M'}\right)^2 + \varepsilon\left(\frac{\Delta\delta_i}{\delta}\right)^2\right]$$
$$\approx N_V\left(\frac{\Delta M}{M}\right)^2 + x(1-x)\left(\frac{M_{Ge}-M_{Sb}}{M'}\right)^2$$

Here, the first term is from vacancy. N_V is the relative vacancy concentration and can be extracted from carrier concentration. Last term (Γ_{Strain}) arises from strain field scattering effect and for the current purposes we can consider it to be negligible as radius of dopant and substituted atom is very similar. Vacancy contribution can be measured by considering formalism adopted from Klemens:[46] $\frac{\Delta M}{M} = -\frac{M_a}{M} - 2$. For vacancy only M is the average mass per atom of Ge and Te group. M_a is mass of missing atom. M' is the average mass of ternary cluster.

$$\Gamma \approx N_V\left(-\frac{M_a}{M} - 2\right)^2 + x(1-x)\left(\frac{M_{Ge}-M_{Sb}}{M'}\right)^2$$
$$A \approx \frac{V}{4\pi v_s^3}\left[N_V\left(-\frac{M_a}{M} - 2\right)^2 + x(1-x)\left(\frac{M_{Ge}-M_{Sb}}{M'}\right)^2\right]$$
$$B_U \approx \frac{\hbar\gamma^2}{M v_s^2 \theta_D}$$

Here, $V = 26.583 \times 10^{-30}\ m^3$ volume per atom. $\gamma = 1.7$ [58]

$$M_a = M_{Ge} = 72.63\ amu$$
$$M = \frac{M_{Ge}+M_{Te}}{2} = \frac{72.63 + 127.60}{2} = 100.115\ amu$$
$$M' = (1-x)M_{Ge} + xM_{Sb} + M_{Te}$$
$$M' = 0.92 \times 72.63 + 0.08 \times 121.76 + 127.60 = 204.1604\ amu$$

$$\left(-\frac{M_a}{M}-2\right)^2 = \left(-\frac{M_{Ge}}{M}-2\right)^2 = \left(-\frac{72.63}{100.115}-2\right)^2 = 7.43$$

$$\left(\frac{M_{Ge}-M_{Sb}}{M'}\right)^2 = \left(\frac{72.63-121.76}{204.1604}\right)^2 = 0.0579$$

1) Pristine GeTe

$N_V = n_p V = 8.04 \times 10^{26} \times 26.583 \times 10^{-30} = 0.0214\, m^{-3}$, $v_s = 1900\, m/s$ [35]

$$A \approx \frac{V}{4\pi v_s^3}\left[N_V\left(-\frac{M_a}{M}-2\right)^2\right] = \frac{26.583 \times 10^{-30}}{4\pi \times 1900^3} \times 0.0214 \times 7.43 = 4.90 \times 10^{-41} s^3$$

$$B_U \approx \frac{\hbar\gamma^2}{M v_s^2 \theta_D} = \frac{1.0544 \times 10^{-34} \times 1.7^2}{100.115 \times 1.66 \times 10^{-27} \times 1900^2 \times 181} = 2.81 \times 10^{-18}\, sK^{-1}$$

2) Doped GeTe

$$N_V = n_p V = 4 \times 10^{26} \times 26.583 \times 10^{-30} = 0.0106\, m^{-3}$$

$$A \approx \frac{V}{4\pi v_s^3}\left[N_V\left(-\frac{M_a}{M}-2\right)^2 + x(1-x)\left(\frac{M_{Ge}-M_{Sb}}{M'}\right)^2\right]$$

$$= \frac{26.583 \times 10^{-30}}{4\pi \times 1900^3} \times [0.0106 \times 7.43 + 0.08 \times 0.92 \times 0.0579]$$

$$= 2.57 \times 10^{-41} s^3$$

$$B_U \approx \frac{\hbar\gamma^2}{M v_s^2 \theta_D} = \frac{1.0544 \times 10^{-34} \times 1.7^2}{102.0802 \times 1.66 \times 10^{-27} \times 1900^2 \times 181} = 2.76 \times 10^{-18}\, sK^{-1}$$

$N_s = 8.9 \times 10^6\, /m$ (microstructure, Fig. 1 e-i), $a_{lat} \sim 4.17 \times 10^{-10}\, m$ (Fig. S2)

$$\tau_{SF}^{-1} = 0.7\frac{a_{lat}^2 \gamma^2 N_s \omega^2}{v_s} \Longrightarrow B_S = 0.7\frac{a_{lat}^2 \gamma^2 N_s}{v_s}$$

$$= 0.7\frac{(4.1719 \times 10^{-10})^2 \times 1.7^2 \times 8.9 \times 10^6}{1900} = 1.6465 \times 10^{-15}\, s$$

Table S1: Fitting parameters used in Callaway's model, where *A* is the point defect scattering parameter, and B_U is three-phonon Umklapp scattering parameter and B_{SF} is the scattering parameter due to stacking faults, respectively. It should be mentioned that B_U essentially remains unchanged regardless of the inclusion or omission of B_{SF}. Moreover, as expected the values of *A* decreases when phonon scattering from stacking fault is also included (i.e., B_{SF} is not = 0). Since a model fit to the experimental data with least number of fitting parameters is preferred, in the main manuscript we focused our discussion on column 3 in Table S1 (see text for more details).

Scattering parameters	$Ge_{0.92}Sb_{0.08}Te$ (with stacking fault)	$Ge_{0.92}Sb_{0.08}Te$ (without stacking fault)	Calculated
A (s^3)	2.81×10^{-44}	4.33×10^{-41}	2.57×10^{-41}
B_U (sK^{-1})	3.73×10^{-17}	2.24×10^{-17}	2.76×10^{-18}
B_{SF} (s)	6.80×10^{-16}	-	1.65×10^{-15}

Quantifying Anharmonicity of GeTe through temperature dependent Raman Spectroscopy

Bulk GeTe exhibits a rhombohedral structure (*R3m* space group with 2 atoms per unit cell) that undergoes a displacive phase transition to a cubic (*Fm-3m*) structure at ~ 673 K. It has 3 vibrational modes that can be represented as $\Gamma = 2E_{2g} + A_{1u}$. They are both Raman-active and IR-active because of the lack of inversion center and their eigenmodes are depicted in the figure below.[59],[60] We employed temperature-dependent polarized Raman spectroscopy data with z(xx)z and z(xy)z configurations from Ref [34].

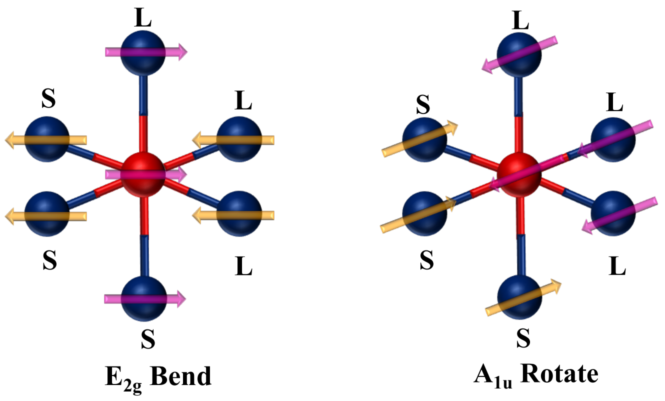

Figure S8a: Raman eigen modes of GeTe.

The anharmonicity co-efficient ($\alpha_{R,j}$) for each Raman mode (ω_j) is given by[26],[61]

$$\alpha_{R,j} = -\frac{3nk_B}{E_T}\left[\gamma\left(\frac{\Delta V}{V}\right) + \left(\frac{\Delta\omega_j}{\omega_j}\right)\right] \qquad \text{(S1)}$$

where n is number of atoms in one mole of the GeTe, $\gamma = 1.7$ is Gruneisen parameter of GeTe[58] E_T is thermal or vibration energy. The experimentally determined volume as a function of temperature is fitted using the following expression.[62]

$$V = V(0) + 9aR\int_0^T dT'\left(\frac{T'}{\theta_D}\right)^3 \int_0^{\theta_D/T'} \frac{e^x x^4}{[e^x - 1]^2} dx \qquad \text{(S2)}$$

where, $\theta_D = 181\ K$ is the Debye temperature of GeTe, that yields, $a = 0.000118\ \text{Å}^3/J$ and $V(0) = 159.509\ \text{Å}^3$ (Fig. S7b).

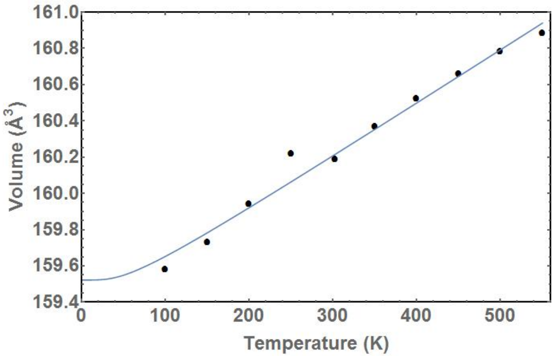

Figure S8b: Volume of GeTe unit cell as a function of temperature. The solid bullets represent experimental data and the fit through the data is described by equation (S2).

Using the extracted values of a and $V(0)$ in equation (S2), we obtained $\Delta V = V(550) - V(0) = 1.429\text{Å}^3$.

The experimental data for temperature dependent polarized Raman E_{2g} and A_{1u} modes from Ref. [34] were fitted (Fig. S8c) using identical three and four phonon decay approximation as,

$$\omega_j(T) = \omega_{oj} + \Delta\omega_j(T)_V + \Delta\omega_j(T)_{anh} \quad \text{(S3)}$$

where, ω_{oj} is the temperature independent harmonic frequency of the jth mode, and $\Delta\omega_j(T)_V$ and $\Delta\omega_j(T)_{anh}$ are the frequency shifts corresponding to quasiharmonic volume expansion and anharmonic phonon-phonon coupling. The qusiharmonic volume expansion contribution to frequency shifts is given by,

$$\Delta\omega_j(T)_V = \omega_{oj}\left[exp\left(-\gamma_{jT}\int_0^T \beta_T(T')dT'\right) - 1\right] \quad \text{(S4)}$$

where, $\beta_T = (1/V)\, dV/dT$ is volume expansion coefficient.

The shift in the phonon frequency due to anharmonic phonon-phonon interaction is given by Eq. (S5), wherein a phonon of frequency ω_j decays into two $(\omega_m, m = 1,2)$ and three $(\omega_n, n = 1,2)$ phonons respectively,[63]

$$\Delta\omega_j(T)_{anh} = A\left[1 + \sum_{m=1}^{2}\frac{1}{e^{\hbar\omega_m/k_BT} - 1}\right] + B\left[1 + \sum_{n=1}^{3}\left(\frac{1}{e^{\hbar\omega_n/k_BT} - 1} + \frac{1}{\left(e^{\hbar\omega_n/k_BT} - 1\right)^2}\right)\right]$$

(S5)

We used identical phonon decay mode for three phonon process, whereas for four phonon process, with the help of phonon dispersion relation and phonon DOS,[31] a decay channel of $160\ cm^{-1} + 86cm^{-1} \leftrightarrows 123\ cm^{-1} + 124\ cm^{-1}$ out of many decay channels available by considering higher phonon is used to fit (Fig. S8c) the temperature dependent shifts in the frequency of both E and A modes. Two 124 cm^{-1} and 160 cm^{-1} optical phonons located in the L-W direction of phonon dispersion and 86 cm^{-1} and 123 cm^{-1} phonons are located at zone center gamma point were selected so that the decay mode satisfy both energy and momentum conservation. Interestingly, we found that the phonon decay in GeTe is dominated by a four-phonon process, unlike SnSe, where three phonon decay is the dominant mechanism. This is because GeTe exhibits a larger phonon energy gap as compared to SnSe where there were low hanging optical modes present.[45]

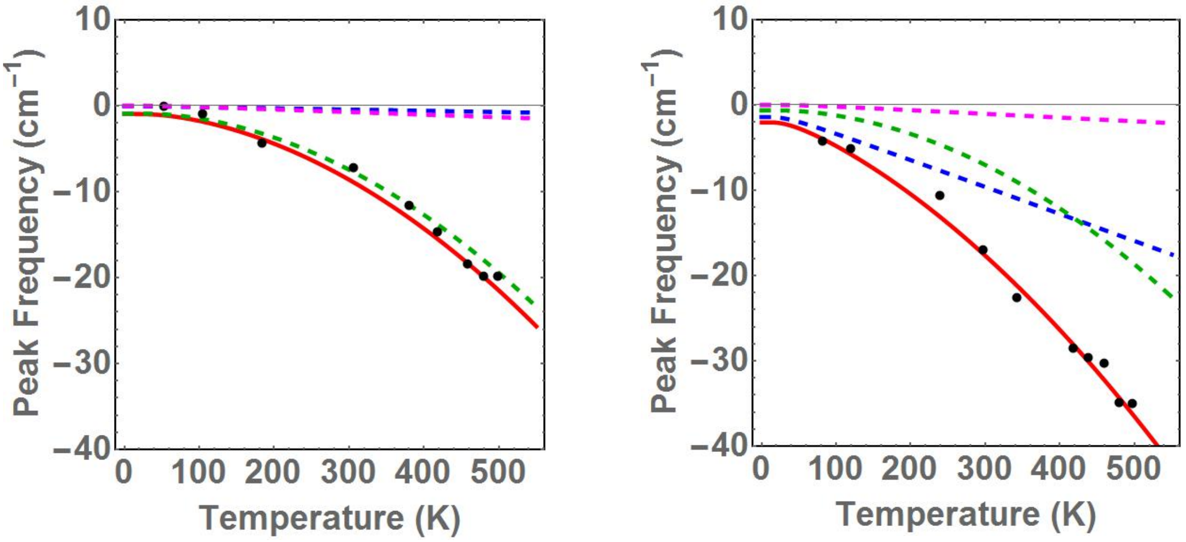

Figure S8c: Frequency shift plotted as a function of temperature for E_{2g} (left panel, 86 cm^{-1}) and A_{1u} (right panel, 123 cm^{-1}) modes. The 3-phonon (blue), 4-phonon (green), thermal expansion (magenta) contributions to the total shifts (red) are shown in the figure.
